# Observation of Ag Nanoparticles in/on Ag@MIL-100(Fe) Prepared Through Different Procedures

**DOI:** 10.3389/fchem.2019.00686

**Published:** 2019-10-22

**Authors:** Rubén Mahugo, Alvaro Mayoral, Manuel Sánchez-Sánchez, Isabel Diaz

**Affiliations:** ^1^Instituto de Catálisis y Petroleoquímica (ICP), CSIC, Madrid, Spain; ^2^Center for High-Resolution Electron Microscopy, School of Physical Science and Technology (SPST), ShanghaiTech University, Shanghai, China

**Keywords:** Ag@MIL-100(Fe), MOF, impregnation, STEM/HAADF, TEM

## Abstract

Loading of active metals, metal clusters, and/or metal nanoparticles in Metal Organic Frameworks (MOFs) is an emergent field with applications in sensors, catalysis, medicine, and even in the polymeric industry. In the present work, MIL-100(Fe) has been synthesized and reacted with AgNO_3_ through liquid and incipient wetness, and also through solid-state reaction or solid grinding. The aim of this study is to evaluate whether the MIL-100 would uptake metal particles using a similar principle as that of the ion exchange in zeolites, or else, their inherent humidity would favor the “dissolution” of the metal salt, thus yielding very small metal particles. The immobilization of Ag nanoparticles inside the MOF pores was identified by C_s_-corrected scanning transmission electron microscopy (C_s_-corrected STEM) techniques.

## Introduction

The study of porous materials is one of the main disciplines in material science, due to their industrial and potential applications, which have even increased in the last few decades (Hermes et al., 2005). Metal Organic Frameworks (MOFs) are the most recent family of ordered porous materials formed by metal nodes connected by organic linkers. These particular building units increase the possibilities of generating new materials with different pore sizes, shapes and topologies that, together with their vast framework chemistry, facilitate the versatility in applications as gas storage (Orcajo et al., 2012; Dutta et al., 2018), catalysis (Wang et al., 2019), sensors (Peng et al., 2016; Bagheri et al., 2018), or redox processes (Moon et al., 2005), among others (Li et al., 2016). Our interest in MOFs relies on the use of facile synthesis methods with low energy and reagents demand. Using this approach, we have obtained highly crystalline and small particle MOFs, suitable for catalytic processes (Díaz-García et al., 2014; Getachew et al., 2014; Ruano et al., 2015; Sánchez-Sánchez et al., 2015; Guesh et al., 2017). The immobilization of metals in MOFs brings a new challenge in supported catalysts (Dhakshinamoorthy and García, 2012), not only in terms of the immobilization efficiency but also in the identification of the metal cluster trapped in the porous network (Houk et al., 2009; Esken et al., 2010; Meilikhov et al., 2010). In this sense, and mainly due to the negative charges introduced by the Al units into zeolite frameworks, the presence of exchangeable cations have facilitated the metal-incorporation chemistry of zeolites that have been dominating this field (Mayoral and Anderson, 2007; Mayoral et al., 2016). Typically, metals are aimed to form nanoparticles or clusters (Mayoral et al., 2011, 2013; Corma et al., 2013; Moliner et al., 2016; Wang et al., 2016) with controllable size, morphology, and composition; however, their trend is to sinter in reactive atmospheres, such as oxidative or reducing environments, humidity, variable temperature, light, or even air. In order to be able to increase the activity, the life-time and therefore the efficiency of the metal, the confined certain space of the MOF pores can be used to control the size of the clusters or nanoparticles and to locate them along the MOF.

Among the many characterization techniques, Transmission Electron Microscopy (TEM) is one of the most adequate to characterize these materials, particularly metal-containing porous solids where the metals are not periodically distributed over the framework. Using TEM, it is possible to characterize both the framework and the guest species by high-resolution imaging combined with electron diffraction, as well as spectroscopic measurements to obtain chemical information. In the particular case of MOFs, the poor stability under the electron beam makes their study significantly complex as they disintegrate under conventional irradiation in just a few seconds (Mayoral et al., 2015, 2018; Zhu et al., 2017; Zhang et al., 2018). Despite these difficulties, we have been able to show evidences of metal cluster trapped inside the porosity of MIL-100(Fe) using spherical aberration corrected (C_s_-corrected) scanning transmission electron microscopy (STEM) in low dose conditions (Mayoral et al., 2017), coupled with an annular dark field detector (ADF). By carefully controlling the e-beam dose, it is not only possible to acquire high-resolution information, but even the formation of metal nanoparticles or even nanowires can be produced (Mayoral and Anderson, 2007; Mayoral et al., 2011). Furthermore, various metals have been supported over different MOFs using different methods (Dhakshinamoorthy and García, 2012). The main challenge in this case remains in obtaining clusters or particles below 5 nm, taking advantage of the flexibly and mobility offered by the porous network of MOFs.

We have focused our study on the incorporation of silver nanoparticles within MIL-100(Fe), because it contains meso-sized cavities and it can be prepared with small particle size and high crystallinity. MIL-100(Fe) is formed by cavities large enough to immobilize Ag inside its pores (Horcajada et al., 2007). Silver was selected because of its catalytic capacity and its application in different fields (Dadashi et al., 2018). Different loading approaches can be beneficial, depending on the required synthesis conditions, and/or on the targeted materials. Although impregnation methods are the most common technique (Rösler and Fischer, 2015; Agundez et al., 2018), solid grinding has resulted into the formation of 2 nm gold metal nanoparticles in certain MOFs (Peng et al., 2016).

In this study, silver nanoparticles have been immobilized over MIL-100(Fe) through different techniques: solid grinding or solid-state reaction, incipient wetness, and ion exchange liquid impregnation. The resulted materials have been studied by different characterization techniques and more in depth by C_s_-corrected STEM as a tool to obtain direct information of the nanoparticle distribution within the support. Indeed, this technique has proved to be the most powerful one to evaluate which method yields to the desired Ag@MIL-100(Fe) composite material.

## Materials and Methods

### MIL-100(Fe)

MIL-100(Fe) was synthesized at room temperature following the procedure developed in our previous work (Guesh et al., 2017). In a first solution, 1.68 g of trimesic acid H_3_BTC (8 mmol, Sigma-Aldrich) was dissolved in 26.9 mL of 1 M NaOH under agitation for 30 min. A second solution was prepared by dissolving 2.38 g FeCl_2_·4H_2_O (12 mmol, Sigma-Aldrich) into 97.2 mL of water. This second solution was added dropwise into the first one, and the resultant mixture was allowed to react under stirring for 24 h. The molar composition of the mixture was 1.0 H_3_BTC : 1.5 Fe : 3.48 NaOH : 860 H_2_O. Afterwards, the solid obtained was recovered by centrifugation and washed three times with excess of deionized water.

### Anionic sod-ZMOF

Anionic sod-ZMOF was prepared following the reported literature (Calleja et al., 2010) but carrying out the crystallization under microwaved-assisted (computer-controlled Milestone ETHOS ONE microwave equipment) heating, in order to reduce the crystal size of the material to make both the ion exchange procedure and their characterization easier by means of transmission electronic microscopy. 4,5-Imidazoledicarboxylic acid (H_3_ImDC) was used as a linker, In(NO_3_)_3_·xH_2_O as metal source, dimethylformamide (DMF) and acetonitrile (CH_3_CN) as solvents, nitric acid (HNO_3_) as modulator, and imidazole (Im) as the structure-directing agent, to give a mixture of the following molar composition: 1 In: 3 H_3_ImDC: 6.9 Im: 24 HNO_3_: 446 DMF: 220 CH_3_CN. This mixture was heated up to 85°C for 12 h (nucleation step) and then up to 105°C for 23 h (crystallization step). The resultant solid was collected by centrifugation and dried at room temperature.

### Impregnation of AgNO_3_

Impregnation of AgNO_3_ was attempted using three methods: Solid state Reaction (SR), Incipient Wetness impregnation (IW), and Ion Exchange in aqueous solution (IE). In all experiments the silver source was AgNO_3_ (Sigma-Aldrich). In the first method, solid-state reaction, a given amount of MOF and AgNO_3_ were ground using mortar and pestle until a homogeneous brown color solid was achieved. Three ratios were tested using Fe: Ag(AgNO_3_) molar ratios of 1:1, 5:1, and 10:1. The samples were labeled SR-1:1, SR-5:1, and SR-10:1. In the case of the incipient wetness impregnation method, the solution volume necessary to reach the incipient point was previously determined using pure distilled water as a adsorbate. Such determined volume of solutions with different AgNO_3_ concentrations were added dropwise to the warm MIL-100(Fe) sample, which had been dehydrated at 180°C for 24 h. The resulting samples were named IW-5:1, IW-10:1 and IW-100:1, according to their corresponding Fe:Ag ratio. The Ion Exchange in aqueous solution was tested for comparison purposes. Aiming similar Fe to Ag ratios, 3 mL of two solutions of AgNO_3_: 0.003M and 0.24M, were tested. The mixtures were stirred for 5 h, and the final solid was recovered by centrifugation. The final samples were named IE-10:1 and IE-100:1. Finally, the anionic sod-ZMOF was ion exchanged under the conditions used for sample IE-10:1 and called IE-SOD. All the samples were heated to 150°C for 12 h following a 2°C/min ramp.

### Characterization Techniques

Powder X-Ray Diffraction (PXRD) patterns were collected in an X-Ray Polycrystalline X'Pert Pro PANalytical diffractometer using CuK_α_ radiation (λ = 1.5406 Å), with accelerating voltage and current of 45 kV and 40 mA, respectively. N_2_ adsorption measurements were conducted at −196°C using a Micrometrics ASAP 2420 sorptometer to determine textural properties. All samples were pre-treated at 150°C for 16 h. Pore size distributions were determined from the adsorption branches of isotherms, using the Barrett-Joyner-Halenda (BJH) following the Kruk-Jaroniec-Sayari model. Scanning electron microscopy and EDX analyses were carried out on a Hitachi TM1000 microscope equipped with EDX detector. Samples were observed without coating. Scanning transmission electron microscopy (STEM) analyses were performed in a cold FEG JEOL GrandARM 300 operated at 300 kV. The microscope is equipped with a double spherical aberration corrector from JEOL. The spatial resolution at 300 kV was 0.7 Å. The microscope is also fitted with a JEOL EDS system and a Gatan Quantum Energy Filter. Typically, the beam current employed was set between 1 and 2 pA, with a total exposure time of 6 s. In prior observation, the samples were crushed using mortar and pestle for 3 min, suspended in ethanol and sonicated. A few drops of the suspension were placed onto holey carbon copper grids.

## Results and Discussion

**Figure 1:**
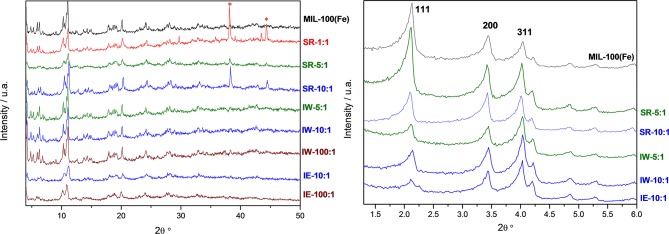
Wide-angle **(Left)** and low angle **(Right)** powder XRD patterns of the Ag-free (top, black line) and Ag@ MIL-100(Fe) samples incorporated by different methodologies and with different Ag loadings. Asterisks at 2θ near 38° and 44° indicate the presence of Ag^0^.

**Figure 2:**
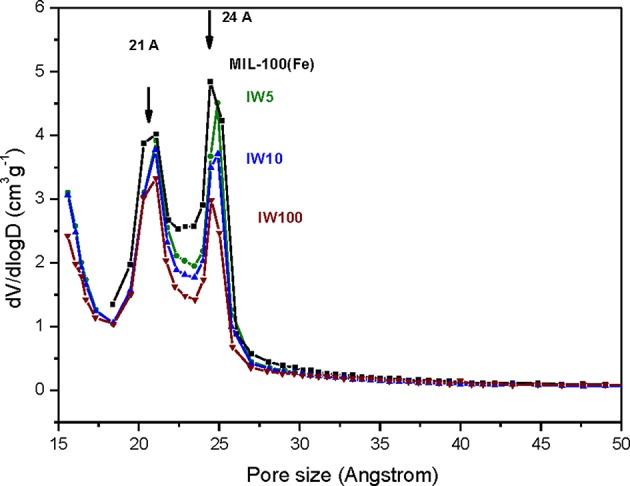
Pore size distribution curves of the pristine MIL-100(Fe) (black) and the different Ag@MIL-100(Fe) samples prepared by wetness impregnation method (IW-5:1, IW-10:1, and IW 100:1).

Metal incorporation within MOF materials opens up new possibilities of use for these materials, as additional properties can be granted increasing their multifunctional behavior. Silver is of particular interest in catalysis, optical devices, and as antibacterial compounds. In order to evaluate the efficiency of the impregnation of silver, a comparison between the different molar ratios was tested. Table 1 collects the different Ag molar ratios contained in the MOF using the three different approaches. Powder X-ray diffraction (PXRD) was used to evaluate the crystallinity of the support prior and after metal incorporation to corroborate if any of the reactions caused any structural damage and if any evidence of metal loading could be extracted. Figure 1-left shows all PXRD profiles of the as-synthesized materials in addition to the pristine MIL-100(Fe), black color. The comparison between all high-angle XRD profiles with that of the Ag-free MIL-100(Fe) evidences that, after the reaction, most of major diffraction peaks were preserved, suggesting that the crystallinity of the MOF prevailed. Nevertheless, it is noteworthy to mention that certain Ag incorporation processes induced a decrease on the general intensity of the diffraction peaks mainly those corresponding to SR-5:1 and both ion exchanged samples. This observation can be attributed either to partial loss of crystallinity or to the successful incorporation of the metals within the pore system. Additional peaks, which do not belong the MIL-100(Fe), were identified at 2θ = 38° and 44°, marked by asterisks in Figure 1-left, that were attributed to Ag^0^ (in particular, they correspond to 111 and 200 reflections, respectively). It is remarkable that these peaks have only been observed in the case of the solid state reaction, suggesting that this approach may incorporate a higher amount of silver, and also that the formation of larger metal isolated particles occurred. Due to the large lattice cell parameters of MIL-100(Fe) a ≈73.34 Å, which falls within the mesoscopic range, low-angle PXRD was also registered (Figure 1-right) as the d-spacing of the 111, 200, and 311 lattice planes are below 4.5° 2θ. It must be noted that such XRD reflections correspond to interplanar d-spacing involving the mesocages of the MOFs (Sanchez-Sanchez et al., 2015; Guesh et al., 2017). In other words, the relative intensity of such reflections, with respect to the intensity of the other reflections found at higher 2θ angle, are very sensitive to the presence of chemical species inside of the cavities, particularly if such species are bulky and formed by heavy atoms, like Ag nanoparticles. The significant changes in the relative intensity between these peaks suggest that some structural transformations (or rather, efficient filling of the mesocages) took place, with special emphasis in the samples SR-10:1, IW-5:1, IW-10:1, and IE-10:1. If the intensity of the low-angle XRD reflections is affected by the presence of Ag species within the MIL-100(Fe) pores, it is expected that textural properties of the Ag@MIL-100(Fe) materials are also modified with respect these of the pristine MIL-100(Fe). Figure SI1 shows the adsorption/desorption N_2_ isotherms of the different Ag@MIL-100(Fe) samples. Regardless of both metal content and metal incorporation method, all Ag-containing samples lost certain pore volume and surface area, which must be attributed to the presence of Ag species in the sample but not necessarily within the MOF pores. Interestingly, the porosity does not vary systematically with Ag content of the samples, suggesting that Ag incorporation by different methods and even different amounts of Ag incorporated by a given method leads to different efficiency in pore filling. Figure 2 compares the BJH pore size distribution (PSD) curves of the MIL-100(Fe) and the different Ag@MIL-100(Fe) samples prepared by wetness impregnation method. The two peaks correspond to the pore diameter of the MIL-100(Fe) meso-sized cavities. These diameters (21 and 24 Å) are slightly smaller than the real ones (25 and 29 Å) due to the well-known underestimation of the mesopore size by the BJH method (Sanchez-Sanchez et al., 2015; Guesh et al., 2017). Ag incorporation to the sample does not modify the position neither the broadening of the PSD peaks, but it entails their intensity decrease, particularly that of the largest cavity, suggesting that Ag species preferably occupied this cavity. Unexpectedly, the intensity decrease is more significant for samples containing less Ag. To deepen this a priori anomalous behavior and the XRD features, we further characterize the samples by means of advanced electron microscopy techniques. Scanning electron microscopy, using backscattered electrons detector, coupled with Energy dispersive X-ray spectroscopy (SEM/EDX) was employed to study the homogeneity of the samples. This mode allows for identifying isolated Ag particles in brighter contrast due to higher atomic number (Figure SI2). The aim of using this technique was to evaluate, at a glance, the formation of isolated Ag-rich particles, i.e., the homogeneity achieved in each loading method, taking advantage of the backscattered electrons detector, which is sensitive to the atomic number. Besides, some rough estimation of the Fe/Ag ratios of the Ag@MIL-100(Fe) particles was taken from EDX spectroscopy. In spite of the fact that EDX in a SEM machine is at most semi quantitative, it is very powerful when it comes to analyzing molar ratios in isolated particles, rather than obtaining a bulk elemental analysis (ICP-OES, for instance) that would not differentiate between the Ag present inside or outside the porous network. The qualitative Fe/Ag ratios given in Table 1 were analyzed over particles showing weaker contrast, that is, over particles mainly formed by the MOF, avoiding brighter particles due to the presence of the isolated/segregated Ag particles. As expected from the PXRD results, samples obtained via solid state reaction, such as SR 5:1 and SR10:1, showed physical mixtures and abundant presence of isolated Ag particles, with very low presence of Ag in the MOF particles, or even below the detection limit. On the other hand, incipient wetness (IW) and ion exchange (IE) gave a very homogeneous distribution of Ag within the MOF crystals, although outer separated small Ag particles could be found in all the samples. Very little difference could be observed in IW-5:1 and IW 10:1 samples, yielding very homogenous and high incorporation of Ag, indicating that this method is very efficient in incorporating higher loadings of AgNO_3_. The same trend is obtained for ion exchange conditions with a clear tendency following the theoretical values. In the sample with low levels of loading 100:1, the presence of Ag is hardly detected being below the detection limit.

**Table 1:** Fe/Ag molar ratios of the Ag@MIL-IOO(Fe) materials prepared by different methods.

**Sample code and molarFe:Ag**	**SEM/EDX Molar Fe/Ag**
SR-1:1	–
SR-5:1	< dect. limit
SR-10:1	< dect. limit
IW-5:1	5
IW-10:1	8
IW-100:1	259
IE-10:1 (0.24 M)	15
IE-100:1 (0.003 M)	97

**Figure 3:**
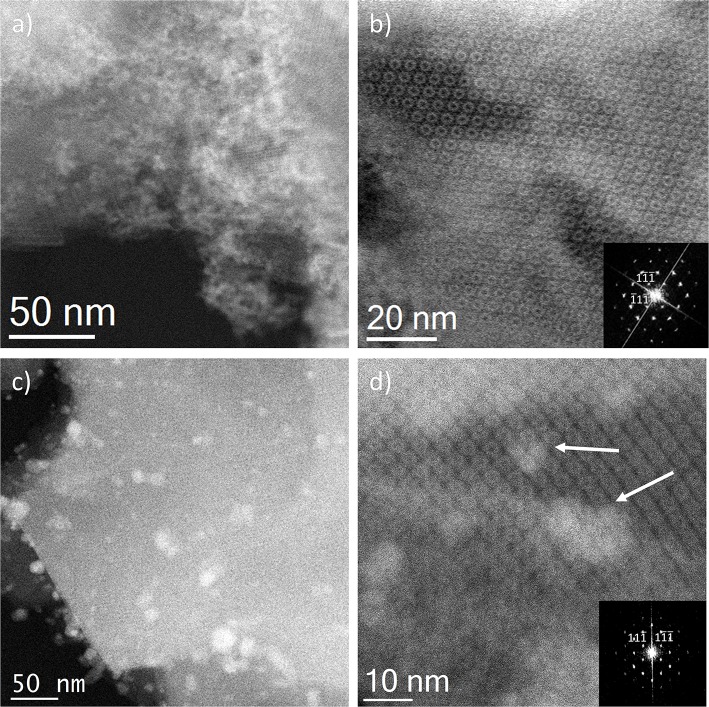
C_s_-corrected STEM-ADF observations for the solid state reactions. **(a,b)** SR-1:1 low magnification and high resolution images, respectively. The Fourier diffractogram (FD) is shown inset of **(b)**. **(c)** Low magnification image of SR-5:1 and **(d)** closer observation of the pore system, where the Ag introduced in the pores is marked by white arrows, the FD inset.

With the intention of further investigating the structure of the support, together with the presence (or absence) of the metal, its shape, its size and its location, TEM analyses were carried out. To do this, spherical aberration corrected (C_s_-corrected) STEM, coupled with an annular dark field detector (ADF), was chosen. This technique has already produced outstanding results on the characterization of beam sensitive nanoporous solids, which, by the use of the ADF detector, also gives direct chemical information as the contrast depends on the atomic number of the elements, thus allowing the clear visualization of the metal with respect to the lighter support. Figure 3 presents the results of the solid-state reaction for the different molar ratios studied. Figures 3a,b show low and high magnification images, respectively, of the samples SR-1:1. In Figure 3a, large domains of Ag (brighter region) with no particular structure, predominant size or morphology on the outer surface of the MOF particles having been clearly identified, as expected from the observation of Ag^0^ peaks in the PXRD (Figure 1). A closer look, Figure 3b, allows clear visualization of the porous framework, proving the good crystallinity of the support after reaction. In this case, a MIL-100(Fe) crystallite is observed sitting on the [110] orientation, which is the most adequate for the visualization of the two types of cavities that form this structure (Mayoral et al., 2017). The contrast variations observed on this projection correspond to the existence of Ag. It is complicated to assess whether Ag is inside, on top or below the MOF. However, the significant changes in the diffraction peaks observed in the PXRD and PSD results, suggest that Ag may be occluded inside the MOF cages, at least to some extent.

**Figure 4:**
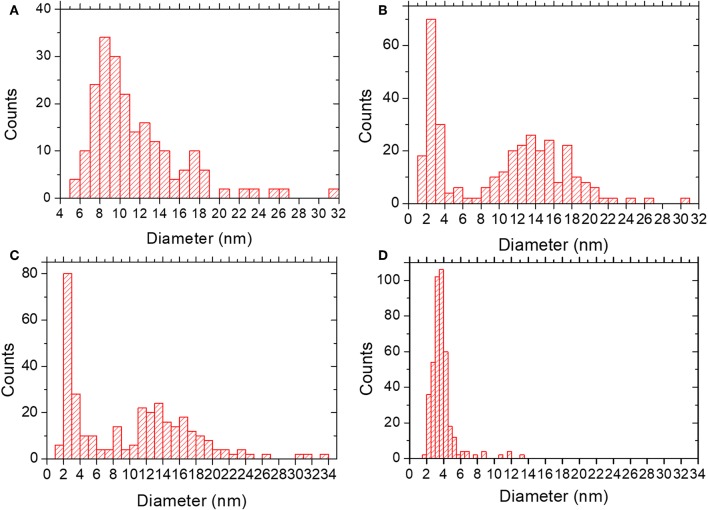
Particle size distribution for **(A)** SR-5:1; mean diameter 12 ± 5 nm. **(B)** IW-10:1; mean diameter of 10 ± 6 nm. **(C)** IE-10:1; mean diameter of 10 ± 7 nm. **(D)** IE-SOD; mean diameter of 4 ± 1 nm.

In the lower metal content sample, SR-5:1, Figure 3c, the low magnification observation reveals the formation of nanoparticles, which are significantly less agglomerated on the surface of the MIL-100(Fe). The mean size obtained was 12 ± 5 nm (see Figure 4A), much larger than the cavities which have diameters of 2.9 and 2.5 nm. Even though silver can be observed outside the MIL-100(Fe), a closer look at the pore system suggests that silver has also gone inside, as the contrast observed matches very well with the porous frameworks (indicated by arrows in Figure 3d). From the comparison of these observations with the PXRD profiles and textural characterization obtained from the solid-state reactions, it can be deduced that crystallinity of the MOF is not affected by the conditions employed here, 210°C. This temperature is below the melting point of the salt and it was chosen to guarantee the preservation of the framework. Under these conditions, the salt should be in solid-state form; however, the presence of the MOF, together with some water that may be allocated in the pores, may induce the melting of the salt at lower temperatures, which is beneficial for its application. Based on electron microscopy observations, it is thus confirmed that the structure did not suffer major alterations and that the diffraction intensity variations in the low angle XRD patterns and N_2_ isotherms owed to the introduction of Ag inside the MIL-100(Fe) cages.

**Figure 5:**
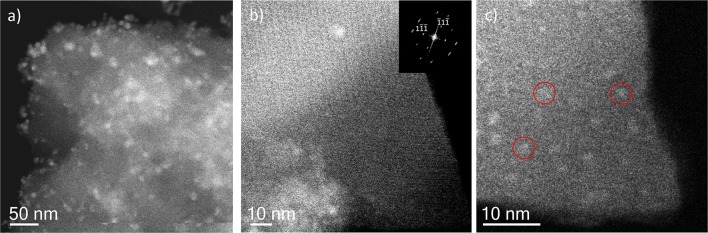
C_s_-corrected STEM-ADF analysis of IW-10:1. **(a)** Low magnification image where several Ag nanoparticles are observed. **(b)** High-resolution image of the framework with the Fourier diffractogram inset. **(c)** A closer look of the edge of the MIL-100(Fe) crystallite where the smallest Ag nanoparticles are marked by red circles.

Incipient Wetness impregnation was also explored as a possibility of Ag incorporation in MIL-100(Fe). In this case, the method requires a low amount of a liquid phase where the salt would be dissolved prior to getting in contact with the dried MOF. As in the previous case, the wide-angle PXRD displayed very good crystallinity after the reaction, which significantly altered some low-angle diffraction peaks and the intensity of the PSD peaks. Figure 5 displays the same analysis as for the SR materials. The samples with different Fe/Ag ratios have been combined in Figure 5, as no differences were found between the 3 samples as expected from SEM. This is in good agreement with the data obtained by PXRD, being the patterns very similar to that of the pristine MOF, except for the systematic changes in the relative intensity at low angle (111, 200, and 311 reflections). Figure 5a presents a low magnification typical image of MIL-100(Fe) where the Ag particles are on the surface. As occurred for the solid-state reaction, for these samples it was also possible to visualize the MIL-100(Fe) network, confirming that the crystallinity was preserved, see Figure 5b. In this image, it is possible to index the FD along the [110] orientation, assuming the *Fd*-3*m* symmetry, see inset. By gently defocusing that image and increasing the electron dose at the cost of certain beam damage, Ag nanoparticles of around 2 nm, smaller than the pore size of MIL-100, are clearly visualized, Figure 5c, marked by red circles, confirming that the MOF materials can act as hosts for the local confinement of silver inside its structure. The analysis of the particle size distribution, Figure 4B, presents and average size of 10 ± 6 nm, similar to the solid-state reaction. However, in this case, two clear domains are identified: (i) one population centered at ≈2.5 nm, and (ii) the second one at ≈13 nm, which is in good agreement with the high-resolution electron microscopy observations.

**Figure 6:**
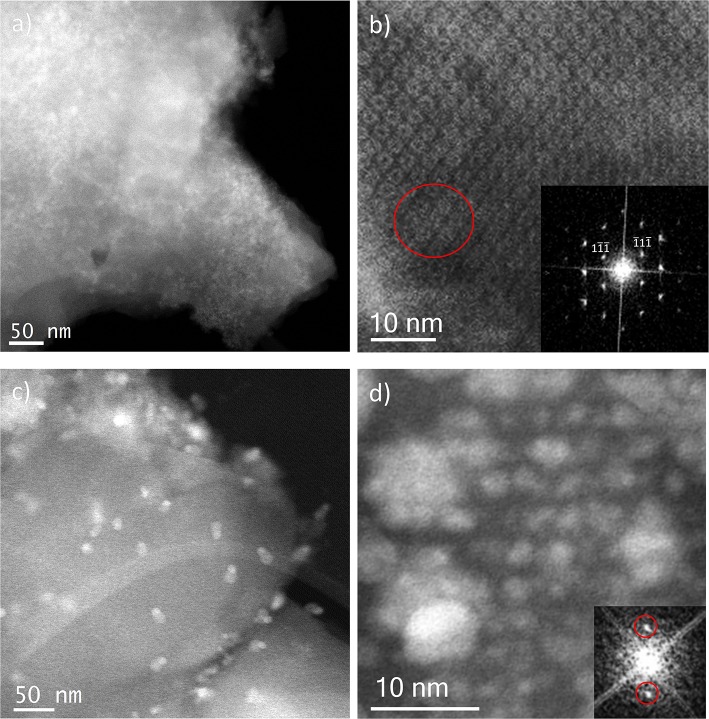
C_s_-corrected STEM-ADF analysis of: **(a)** IE-100:1 low magnification image and **(b)** IE-100:1 high-resolution micrograph along the [110] orientation with the FD shown inset; and **(c)** IE-10:1 low magnification image where the large Ag nanoparticles can be observed and **(d)** IE-10:1 closer observation of a MIL-100(Fe) crystal with the small Ag nanoparticles in a perfect array that matches with the pore system. The FD obtained from that metal distribution is shown inset.

Ion Exchange in aqueous solution (IE) was the third type of metal incorporation method studied in this work. The relative intensity of the diffraction patterns has decreased in comparison to the parent MOF but still retains a good degree of crystallinity. The low angle analysis also shows clear evidence of structural changes by the decrease in relative intensity of the 111 diffraction peak in comparison with the 200 and 311, which may suggest once again that metal is present inside the porous network. As occurred for the wet impregnation, very similar PXRD results were obtained for the different Fe/Ag ratios. Figure 6a depicts a typical image of the IE-100:1, where it is possible to observe the presence of Ag due to its higher contrast, but with no defined morphology. It seems that for this low amount of silver, the metal may form very small sized nanoparticles embedded in the structure. The high-resolution image along the [110] orientation is shown in Figure 6b, where the contrast variations are owed to the presence of the Ag within the pores. Figure 6b shows that Ag occupies the cages and that adjacent cages could be filled with metal, giving the impression of larger formations as indicated by the red circle.

Sample IE-10:1 contains 10 times higher Ag loading, which was evident from the STEM observations. Figure 6c presents the low magnification micrograph, clearly showing the formation of Ag nanoparticles on the surface of the MIL-100(Fe). The average particle size was estimated in 10 ± 7 nm, with two populations clearly identified. A narrow distribution centered around 2.5 nm and a second wider one of ~13 nm (Figure 4C) resembling what was observed in the samples prepared by the wet impregnation method. Interestingly, for this material it was also observed how the small nanoparticles, which would fit the pore size, entirely filled the MOF crystal as depicted in Figure 6d. It was observed how 2 nm Ag nanoparticles were periodically aligned compacted in a MIL-100(Fe) crystallite, corroborating the confinement effect thanks to the introduction of the metal inside the cages. The Fourier diffractogram corresponding to that array is shown inset, where the diffraction spots are denoted by red circles situated at a distance of ≈ 37.20 Å, which would correspond to the d-spacing of the {200} planes MIL-100(Fe).

**Figure 7:**
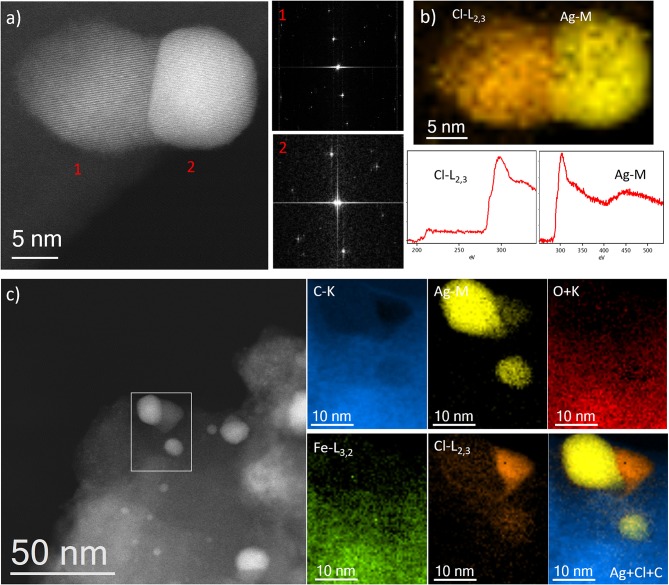
**(a)** Cs-corrected STEM-ADF image showing a particle with two different regions marked as 1 and 2. The FDs are also shown to the correspondent regions. **(b)** EEL spectrum imaging with Cl as orange and Ag as yellow, the correspondent spectra of each edge are shown below. **(c)** Cs-corrected STEM-ADF image where the area of analysis is marked by a white rectangle. The different compositional maps are presented separately. Additionally, the Ag, Cl, and C map is also shown.

A very particular aspect only observed for the sample IW-10:1, with sufficient amount of Ag, was the morphology of the nanoparticles situated on the surface of the MOFs. In many cases, different contrast was appreciated within the NPs. Keeping in mind that the contrast is Z dependent, it needs to be explained in terms of different composition. Figure 7a shows the high-resolution image of a typical example, where the nanoparticle is composed by two regions that crystallized in different structures, as confirmed by the high-resolution image and by the FDs. Indexing the FD of the lower contrast region gives an interplanar distance of 3.16 Å in agreement with the d_111_ planes of AgCl obtaining a unit cell value of a = 5.47 Å. The FD of the brightest area numbered as 2, can be indexed as pure Ag on the [110] projection obtained a d_111_ of 2.35 Å to give a lattice constant of a = 4.07 Å. The chemical analysis performed by EELS, Figure 7b, corroborated the brightest region correspond to pure Ag, yellow map, while the lighter one is formed by AgCl, orange color. Both spectra, corresponding to the Cl-L_2, 3_ and the Ag-M signals, are presented in the bottom part of Figure 7b. Further chemical spectra imaging was recorded over a larger region (Figure 7c) displaying similar results.

Every studied method has successfully introduced Ag in different forms in the pores of MIL-100(Fe), and they have also resulted in the formation of small nanoparticles on the surface of the porous materials. In this aspect, although some nanoparticles were outside the framework, their formation were also affected by the presence of the porous network. In the solid-state reactions, a high content of salt yielded the introduction of Ag in many cages but with no particular morphology. This is the case of sample SR-1:1 where the excess of Ag was spread over the porous particles, even in some regions on their surface. Among the possibilities of how metals were introduced into the framework, cations may be transferred through vapor phase or surface diffusion (Karge, 2008). In our case, surface diffusion may be the process that took place as the temperature used did not even reach the melting point of the salt and, therefore, an intimate contact between the salt in its molten form and the MOF, may favor the introduction of the metal within the pores followed by the self-reduction of Ag at this temperature in the presence of light. In the samples with higher loading, Ag seems to occupy many contiguous pores and that is the reason for the contrast variations within the framework. Furthermore, due to the high salt content, not all the salt penetrated into the system and larger domains of non-crystalline Ag^0^ were formed. When Ag concentration decreased, the process occurred in a similar way and the MIL-100(Fe) facilitated the diffusion of the metal along its porosity, allowing Ag to nucleate, forming Ag NPs (nanoparticles) whose size may be restricted by the Ag reservoir. In this case, the available Ag is significantly lower in comparison with the sample SR-1:1, so well-defined Ag nanoparticles were formed, in many cases on the surface of crystallites.

As a general trend, the two cases involving liquid media, wet impregnation and liquid ion exchange IE-(10:1), produced similar results. The dissolved Ag, forming separated Ag^+^ cations would enter in the pores and nucleate as Ag clusters/nanoparticles that fill certain pores. In addition, owing to the high mobility of silver along the framework, silver nanoparticles were found on the surface of the MOF particles. In this case, some Ag^+^ may migrate onto the surface where it began to nucleate forming larger particles, as no structural constrains were present; these two types of Ag entities were made clear through the particle size analysis, which presented a bimodal behavior in both cases: (i) the formation of one family of nanoparticles of size around 2.5 nm that would fit the pore cages of MIL-100(Fe), and (ii) the second one with much larger size (of ca. ≈13 nm of diameter) that was observed outside the framework. This indicates that MIL-100(Fe) structure limits the size of the Ag clusters growing in its pores, as the outside Ag trends to form (around five times) larger particles.

The excess of water in the ion exchange solution assured that silver cations are separated from their counter ions and this aspect facilitated their in-going process; in this case the cavities acted as confined space limiting the formation of larger nanoparticles and allowing the formation of an array of nanoparticles within the pores. In fact, this sufficient presence of separated silver cations also derivate into their reaction with Cl^−^, whose origin must be related to residual counter ion from the Fe source (FeCl_2_) used in the synthesis of MIL-100(Fe).

**Figure 8:**
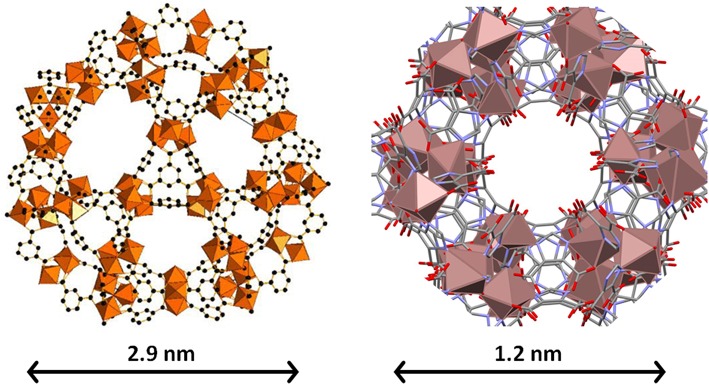
The biggest cages in both MIL-100(Fe) **(Left)** and sod-ZMOF **(Right)** materials. Note that they are at different scales, which is evidenced by the different scale bar and by the size of the metal (Fe and In, respectively) polyhedra. The inner diameter of the cavities is 2.9 and 1.2 nm, and they are accessible through windows with apertures of 0.86 and 0.41 nm. In the case of sod-ZMOF, the compensation cations have been omitted to highlight the porosity.

**Figure 9:**
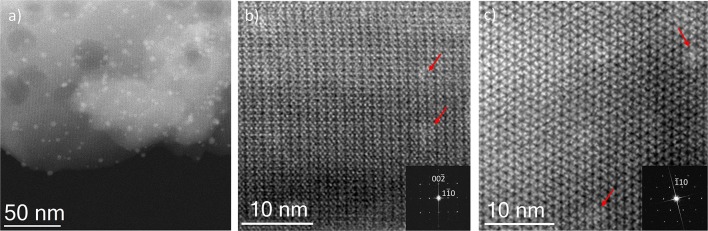
C_s_-corrected STEM-ADF observation IE-SOD MOF. **(a)** Low magnification micrograph showing the presence of Ag NPs. **(b)** Closer observation on the [110] orientation, where some pores are filled with Ag. The FD is shown inset indexed in the *Fd-*3*c* space group. **(c)** High resolution data on the [111] that also confirms the presence of Ag within the pores, the indexed FD is shown inset.

Finally, for comparison purposes, a charged In-based sod-ZMOF was also tested with the intention of evaluating the additional functionalization through the presence of a negative charge into the framework. Unlike MIIL-100(Fe), sod-ZMOF is a microporous material, lacking any mesoporosity (Liu et al., 2006; Calleja et al., 2010). Figure 8 shows the most representative cavities of both MOFs. From correctly interpreting this figure, it is clear that the scale of the pore size is noteworthy, revealing the enormous difference in this feature. The anionic sod-ZMOF material has the topology of the zeolite sodalite and is formed by indium as the isolated (non-clustered) metal node, 4,5-imidazoledicarboxylate as the organic linker and imidazole as structure-directing agent. Its negative charge is compensated by imidazolium in the as-synthesized form. Fortunately, these cations are at least partially exchangeable by more conventional and smaller cations, increasing its available porosity. The condition of anionic framework was expected to favor the attraction of silver cations in a similar manner as occurs for zeolitic materials with Al ions incorporated within the framework. Even single Ag atoms could be potentially formed, favored by the presence of the negatively-charge framework (Liu et al., 2006; Calleja et al., 2010; Chen et al., 2011). A low magnification image of a IE-SOD sample (Figure 9a), after incorporating Ag by ion exchange (IE) procedure, shows the formation of Ag nanoparticles. However, there is a major difference compared with previous experiments. In this case, the particle size is very homogeneous, see Figure 4D, and the diameter is significantly smaller than the former ones with a mean value of 4 ± 1 nm. Such size is quite different to the 13 nm Ag particles formed outside MIL-100(Fe) by the same method, which suggests that chemistry of the MOF surface (composition, defects, charge, hydrophilic character, etc.) could determine the Ag aggregation trend and, therefore, the Ag nanoparticles size, even if they are not formed within their pores. Figures 9b,c display the high-resolution data of the IE-SOD along the [110] and [111] orientations with the corresponding Fourier diffractograms indexed in agreement with its *Fd-*3*c* space group symmetry. These two images allow the visualization of the porous system along with brighter signals that correspond to the Ag occluded inside the pores, marked by red arrows. The narrow pore distribution obtained for this material, together with the small particle size (4 ± 1 nm), can give some hints as to the particle formation mechanism as previously discussed. Clearly, the MOF material acts as a host for the metal during the reaction between the porous material and the metal-containing solution. Eventually, the metal may migrate onto the surface where it begins to nucleate and grow into small nanoparticles. The size of these nanoparticles may be limited by the amount of metal accessible to migrate and the growing process is limited by the amount of metal within the pores. In the case of negatively charged MOFs, more silver was initially occluded into the framework in a more homogenous manner, that allowed the formation of many nucleation points with similar Ag reservoir accessible to them, leading to the formation of many well-distributed metal nanoparticles of relatively small size.

## Conclusions

Different simple methods to incorporate Ag clusters/nanoparticles in a mesoporous MIL-100(Fe) support, prepared under sustainable conditions, have been studied. Some different characterization techniques could provide indirect features of the resultant samples, particularly regarding the Ag particles. However, electron microscopy with the most advanced C_s_-corrected and detector technologies provides direct and reliable information regarding the location of the metal nanoparticles inside the porous network, in spite of the low structural stability of the MOFs under the electron beam. Supported by other valuable techniques, such as XRD, N_2_ adsorption/desorption isotherms and EDX, TEM characterization was able to unequivocally identify both the location and the size of the resultant clusters and/or nanoparticles (or even potentially atoms). Attempts to incorporate Ag by simple solid state reaction of AgNO_3_ with the MOF almost exclusively led to the formation of relatively large and heterogeneously-sized Ag nanoparticles on the external surface of the MOF; scarce Ag was incorporated within the MOF pores. Both wetness impregnation and ion exchange approaches incorporated a significant amount of Ag clusters within the pores, in addition to external Ag nanoparticles, more homogeneous in size than by the solid state reaction approach. The size of such Ag clusters (2.5 nm of diameter with narrow distribution) observed inside of the MOF mesopores (2.5 and 2.9 nm) indicates that the MOF plays an effective role of steric constrain for Ag clusters, which makes MIL-100(Fe) a good candidate to act as a guest of metal nanoparticles in different applications.

Although the here-presented experimental procedures for efficiently creating Ag clusters in MOF pores could be widely improved, this work makes clear that simple experimental modifications can have an important influence in both chemistry and location of the Ag species. More importantly, the advanced TEM techniques developed in this study have shown enormous potential to characterize, in depth and in detail, this particular Ag@MIL-100(Fe) system or, more generally, any metal@MOF system.

## Data Availability Statement

The datasets generated for this study will be made available by the authors, without any undue reservation, to any qualified researcher.

## Author Contributions

All authors listed have made a substantial, direct and intellectual contribution to the work, and approved it for publication.

### Conflict of Interest

The authors declare that the research was conducted in the absence of any commercial or financial relationships that could be construed as a potential conflict of interest.
