# Supplementary material for: Observation of Ag Nanoparticles in/on Ag@MIL-100(Fe) Prepared Through Different Procedures
Source: Front Chem. 2019 Oct 22;7:686. doi: 10.3389/fchem.2019.00686 (PMC6817505; doi:10.3389/fchem.2019.00686)
Supplement: Supplementary file 1 [file Data_Sheet_1.PDF]

Ag-supported metal-organic framework MIL-100(Fe)

Rubén Mahugo,[a] Alvaro Mayoral,[b] Manuel Sánchez-Sánchez,[a] Isabel Díaz[a,]*

[a] Instituto de Catálisis y Petroleoquímica (ICP), CSIC, C/Marie Curie, 2, 28049 Madrid, Spain
[b] Center for High-resolution Electron Microscopy (CħEM), School of Physical Science and Technology ShanghaiTech University, 309 Middle Huaxia Road, Pudong, Shanghai, 201210, China

* corresponding author: idiaz@icp.csic.es

Figure SI1. N_2 adsorption/desorption isotherms at -196 ºC of the Ag@MIL-100(Fe) samples with different Ag contents and prepared by different methods. The determined pore volume and BET surface areas are indicated.

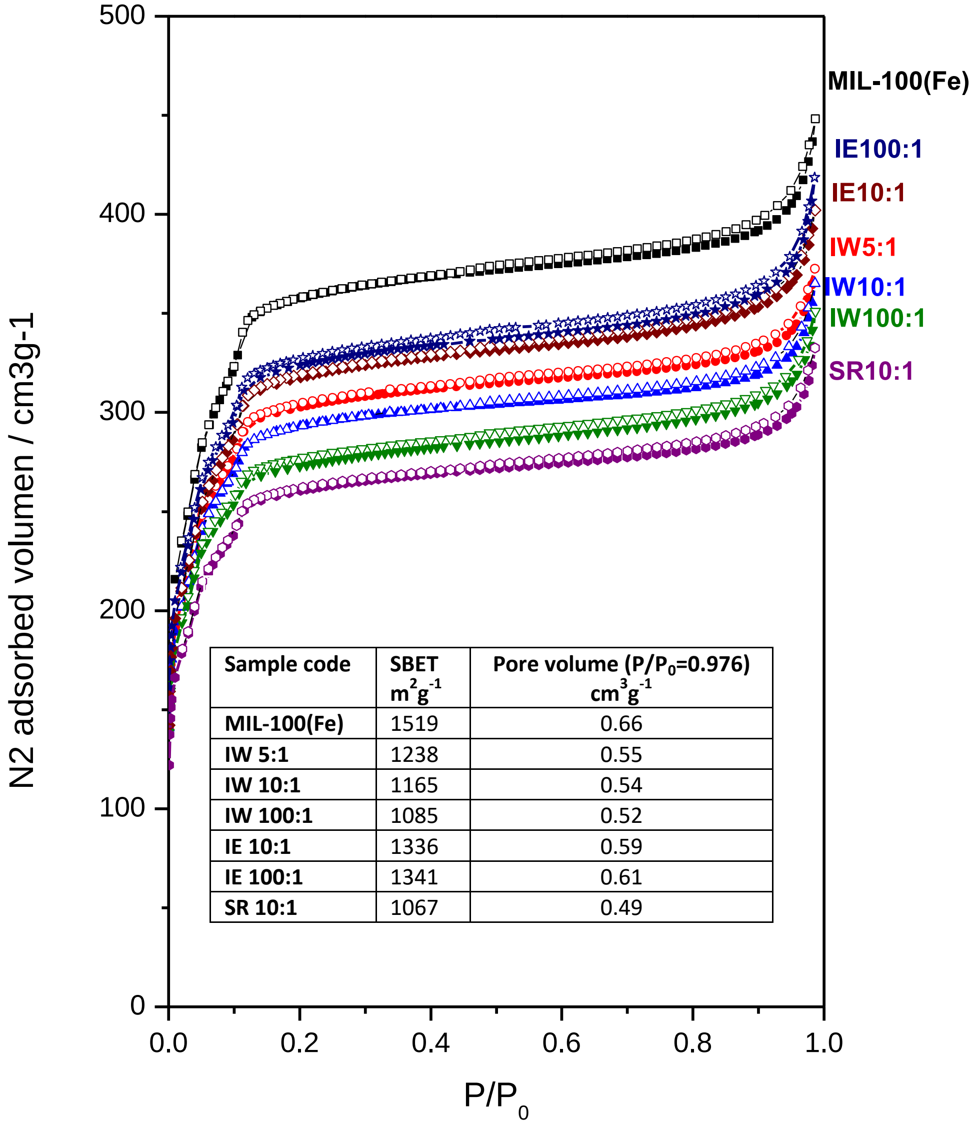

Sample code	SBET m^2g^{-1}	Pore volume (P/P_0=0.976) cm^3g^{-1}
MIL-100(Fe)	1519	0.66
IW 5:1	1238	0.55
IW 10:1	1165	0.54
IW 100:1	1085	0.52
IE 10:1	1336	0.59
IE 100:1	1341	0.61
SR 10:1	1067	0.49

Figure SI2. Representative SEM micrographs of selected samples

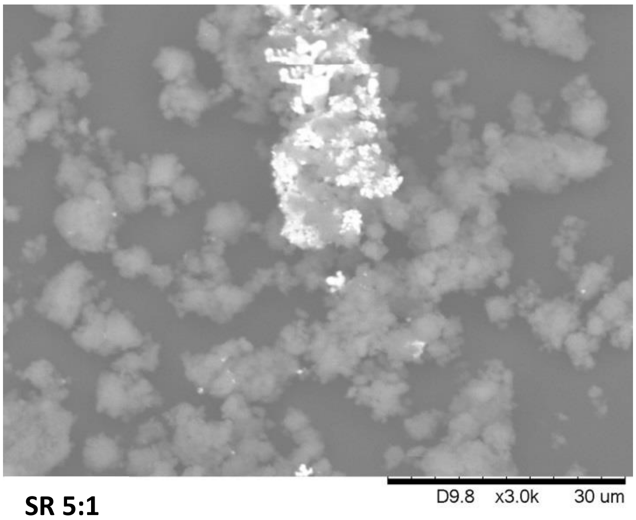

SR 5:1

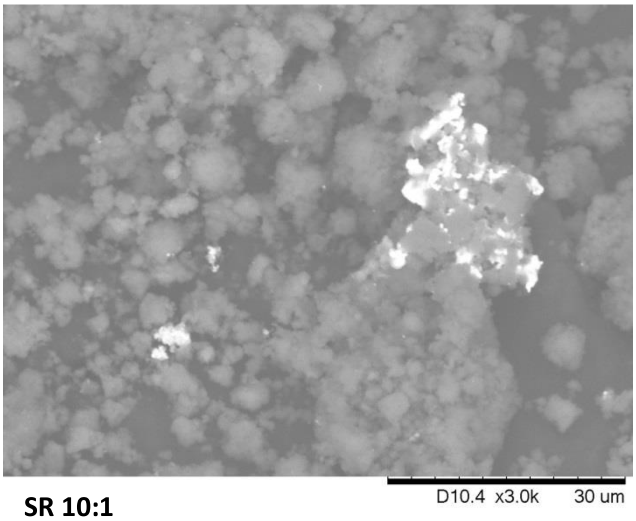

SR 10:1

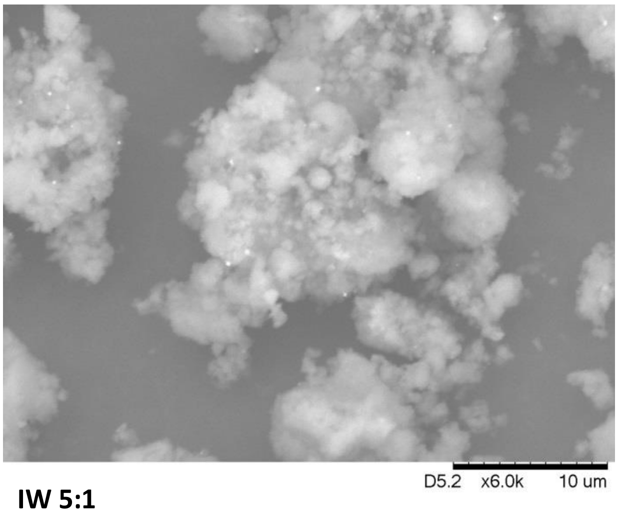

IW 5:1

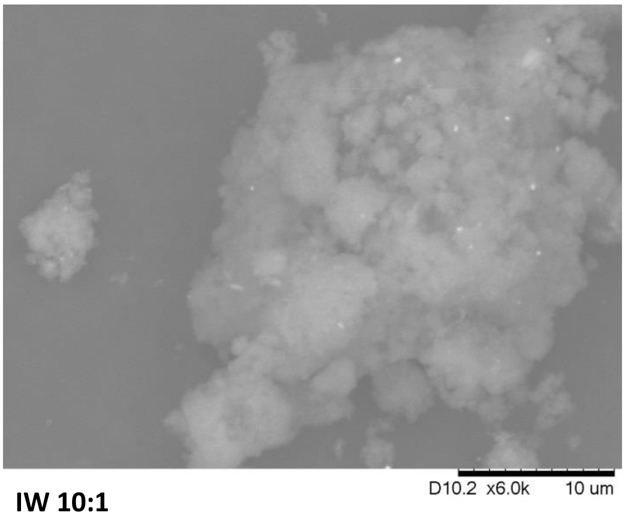

IW 10:1

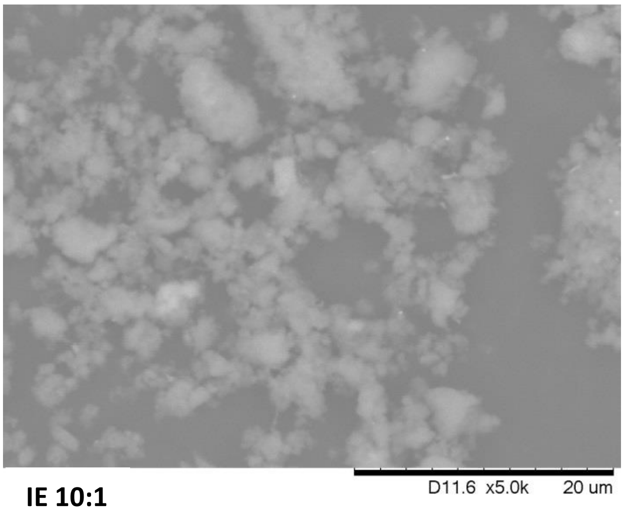

IE 10:1

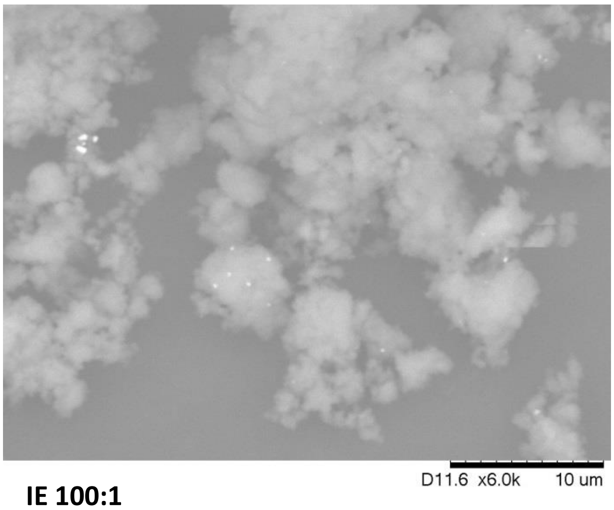

IE 100:1
